# Lipid rafts promote liver cancer cell proliferation and migration by up-regulation of TLR7 expression

**DOI:** 10.18632/oncotarget.11697

**Published:** 2016-08-30

**Authors:** Yuan Liu, Xiaodong Guo, Liyuan Wu, Mei Yang, Zhiwei Li, Yinjie Gao, Shuhong Liu, Guangde Zhou, Jingmin Zhao

**Affiliations:** ^1^ Department of Pathology and Hepatology, Beijing 302 Hospital, Beijing, China; ^2^ First Hepatobiliary Surgery Center, Beijing 302 Hospital, Beijing, China

**Keywords:** lipid rafts, TLR7, proliferation, migration, hepatocellular carcinoma

## Abstract

Hepatocellular carcinoma (HCC) occurs predominantly in patients with underlying chronic liver disease and cirrhosis. Toll-like receptors (TLRs) play an important role in innate immune responses and TLR signaling has been associated with various chronic liver diseases. Lipid rafts provide the necessary microenvironment for certain specialized signaling events to take place, such as the innate immune recognition. The purpose of this study was to determine the pattern of TLR7 expression in HCC, how to recruit TLR7 into lipid rafts responded to ligands and whether targeting TLR7 might have beneficial effects. The study group was comprised of 130 human liver tissues: 23 chronic hepatitis B (CHB), 18 liver cirrhosis (LC), 68 HCC and 21 normal livers. The expression of TLR7 was evaluated using immunohistochemistry, western blotting, and flow cytometry. Proliferation and migration of human HepG2 cells were studied following stimulation of TLR7 using the agonist gardiquimod and inhibition with a specific antagonist 20*S*-protopanaxadiol (aPPD). The activation of lipid raft-associated TLR7 signaling was measured using western blotting, double immunohistochemistry and immunoprecipitation in liver tissues and HepG2 cells. TLR7 expression was up-regulated in human HCC tissues and hepatoma cell line. Proliferation and migration of HepG2 cells *in vitro* increased significantly in response to stimulation of TLR7. TLR7 inhibition using aPPD significantly reduced HepG2 cell migration *in vitro*. The lipid raft protein caveolin-1 and flotillin-1 were involved with enhanced TLR7 signaling in HCC.

**Conclusions:**

The data suggest that inhibiting TLR7 with antagonists, like aPPD, could potentially be used as a novel therapeutic approach for HCC.

## INTRODUCTION

Hepatocellular carcinoma (HCC) is the most frequent primary malignancy of the liver and around 1% of all deaths in the world were related to HCC. In light of this, HCC is one of the cancers with the highest mortality rates worldwide [1–4]. HCC is now the third leading cause of cancer deaths in the Asia-Pacific region [5]. In particular, 55% of the cases worldwide are estimated in China [6]. Due to multiple etiologic factors affect HCC, it makes HCC an extremely complex condition associated with a poor prognosis and its underlying mechanisms remain unknown.

The risk of HCC development is mainly associated with chronic hepatitis B virus (HBV) infection with cirrhosis [7, 8]. The prognosis of HBV patients is closely related to their immune status. The persistence of HBV infection in human liver is believed to be associated to impaired function in activation of type I interferons (IFNs-α/β) [9, 10], a key anti-viral response. This may involve inhibition of plasmacytoid dendritic cells (pDCs) and alterations in natural killer (NK) cell activation. pDCs play an essential role by recognizing foreign pathogens and tumor cells through a panel of pattern recognition receptors (PRR) [9–11], particularly toll-like receptor 7 (TLR7). Upon stimulation of TLR7, they produce and release type I IFNs and other cytokines/chemokines, and cause activation of NK cells and cross priming of cytotoxic lymphocytes, thereby orchestrating both innate and adaptive immune responses.

A variety of chronic liver diseases activate the TLR7 signaling pathway. In turn, TLR7 signaling pathway plays a crucial role in sustained chronic hepatic inflammation [12]. Inappropriate activation of TLR7 can also lead to inflammatory-related cancers. TLR7 initiates the myeloid differentiation response protein 88 (MyD88)-dependent signaling pathway [13]. The ligation of TLR7 and MyD88 results in the phosphorylation of IL1-receptor associated kinase which in turn induces activation of the NFκB pathway. Activation of downstream NFκB pathway initiates the production of pro-inflammatory cytokines such as TNF-α, IL-2, IL-6, IL-8, and especially IFNs-α/β. The activation of the MyD88-dependent TLR7 pathway eventually results in the stimulation of NFκB, which in turn leads to inflammation and carcinogenesis [14, 15].

When TLRs encounter their ligands, activation of TLRs is largely governed by their subcellular localization [13]. The binding of TLR7 and MyD88 results in the assemblies gathering in the particular microdomains of the cell surface, named as lipid rafts, enriching in cholesterol and sphingolipid and functioning as activation platforms for a variety of molecules and participate in many biological processes, such as signal transduction and membrane trafficking [16]. For instance, TLR4 transiently trafficks to lipid rafts after stimulation of macrophages with lipopolysaccharide (LPS), which results in subsequent production of chemokines and inflammatory cytokines [17, 18]. Besides, lipid rafts are also regarded as the requirement for NFκB activation induced by IL-1, LPS, CD40, or CD3/CD28 [19–21], and the destruction of lipid rafts leads to the restraint of NFκB activation induced by these stimuli. Enough these studies show that lipid rafts play an increasingly vital role in the activation of TLR signaling.

In the previous study, we found caveolin-1 and flotillin-1 which were two typical lipid raft resident proteins hyper-expressed in HCC compared to adjacent noncancerous tissues (ANT), and confirmed that lipid rafts played an essential role in HCC [22]. Cholesterol is usually considered acting as a gasket between sphingolipid hydrocarbon chains and functioning as dynamic glue to make lipid rafts gather together [23]. Methyl-β-cyclodextrin (MβCD) can deplete membrane cholesterol and act as a lipid raft disruptor [24]. As a metabolite of ginseng saponins, 20*S*-protopanaxadiol (aPPD), possessing of cholesterol-like structure, can inhibit Akt activity and lead to apoptosis in U87MG cells by interacting with lipid rafts [24]. TLR7 is one of membrane-bound receptors localized intracellularly on endosomal membranes which act as foci for signaling complexes. Dysregulation of endosomal signaling is causal to tumorigenesis [25], and activation or inhibition of TLR7 is also familiar to affect cell proliferation [26]. In addition, excessive expression of TLR7 has been demonstrated in a variety of malignant tumors [27, 28]. As a selective TLR7 agonist, gardiquimod is a new small synthetic antiviral molecule of imidazoquinoline, and its activity is 10 times stronger than imiquimod [29]. Therefore, targeting TLR7 and modulating TLR7 signaling have emerged as a novel therapeutic approach for the prevention and treatment of cancer.

Although the roles of TLRs have been reported in inflammation and cancer, TLR7 in HCC has not been much explored. To gain a better understanding of the role of TLR7 in HCC proliferation and migration, this study focused on TLR7 and HCC associated with the dysregulation of intercellular signaling pathway caused by changes in raft-associated TLR7 expression in the microenvironment of the tumors. Does the increased TLR7 signaling root in accumulated trafficking of TLR7 to lipid rafts? Alternatively, is there a direct relationship among cholesterol content, lipid raft content, and the hyper-responsiveness to TLR7 agonist? Finally, does TLR7-specific agonist also induce increased inflammatory signaling in HCC? For this purpose, we investigated the expression of TLR7 *in vivo* in the liver tissues of HCC patients and *in vitro* in the hepatoma cell line HepG2. Furthermore, we investigated that whether lipid rafts associated with intensified TLR7 signaling in HCC. Finally, we investigated downstream NFκB pathway which involved exaggerated raft-associated TLR7 signaling in HCC.

## RESULTS

### TLR7 expression in human HCC tissues

**Figure 1:**
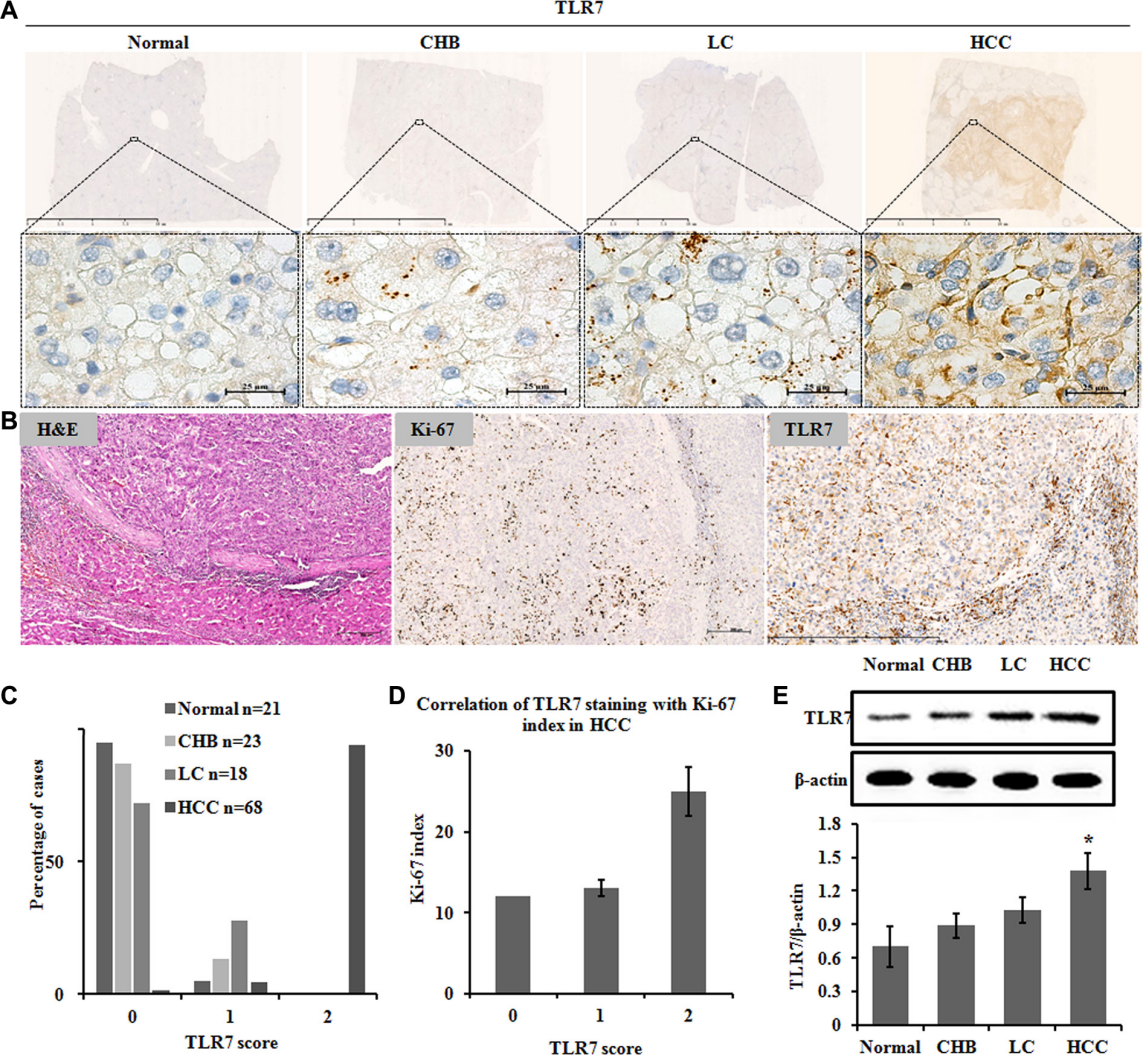
TLR7 expression in human HCC tissues Immunohistochemistry showing (**A**) TLR7 was highly expressed on cell membrane and perinuclei only in malignant hepatocytes but as the scattered spots in cytoplasm of hepatocytes of Normal, CHB and LC. (**B**) Correlation of TLR7 and Ki-67 expression in HCC. Serial sections from the same paraffin block were stained with different antibodies. Hepatic expression of the proliferative marker Ki-67 was high in nuclei. Membranous TLR7 expression was also high in malignant hepatocytes. (**C**) Percentage of cases from the validation set demonstrating no (0), weak (1) or high (2) expression of TLR7. (**D**) Correlation of TLR7 staining with Ki-67 index in HCC. (**E**) Western blotting analysis of TLR7 in liver tissue lysates. Bands were observed at approximately 120 kDa and normalized by β-actin. There was a significant increase in the TLR7 expression of HCC group compared with Normal control. *P < 0.05, results were representative of three independent experiments.

TLR7 was overexpressed in human HCC compared with CHB and LC tissues. TLR7 was predominantly membranous and perinuclear in malignant liver cells but was expressed in the hepatocyte cytoplasm as the scattered spots in normal, CHB and LC tissues (Figure 1A). The percentage of membranous TLR7 expression in HCC was significantly higher than Normal, CHB and LC patients (*P* = 0.001). Specifically, membranous expression of TLR7 in HCC patients was 94.12% but lacking in 95.24% of Normal livers, 86.96% of CHB and 72.22% of LC patients. Faint membranous TLR7 was detected in 13.04% of CHB and 27.78% of LC (Table 1) tissues.

**Table 1:** Tissue clinical data and TLR7 staining

Variables	% TLR7 negative in cytoplasm (*n* = 57)	% TLR7 positive on cell membrane (*n* = 73)	*P*-value
**Age** (mean ± SD)	46.96 ± 10.49	49.51 ± 9.44	
**Sex**			
Female (*n* = 43)	60.47	39.53	0.000
Male (*n* = 87)	35.63	64.37	
**Histologic grade**			
G1 (*n* = 11)	0	100	0.000
G2 (*n* = 48)	6.25	93.75	
G3 (*n* = 9)	11.11	88.89	
**HBV status**			
HBsAg + / HBeAg + (*n* = 50)	32	68	1.000
HBsAg + / HBeAg −(*n* = 59)	35.59	64.41	
**Tissue type**			
Normal (*n* = 21)	95.24	4.76	0.001
CHB (*n* = 23)	86.96	13.04	
LC (*n* = 18)	72.22	27.78	
HCC (*n* = 68)	5.88	94.12	

Membranous TLR7 was observed in 94% of HCC in score 2. Furthermore, membranous TLR7 was observed in 41% of liver cancer with cirrhosis and in 28% of LC patients in score 1 (Figure 1C). In general, the above results suggested that the expression level of membranous TLR7 in HCC patients was significantly higher than that in patients with liver cirrhosis (*P* < 0.001), while membranous TLR7 was rarely expressed in Normal liver.

The expression of TLR7 in the CHB, LC and HCC groups significantly increased compared with the normal group (Figure 1E). A rising trend of TLR7 expression was found in the three patient groups.

### Correlation of TLR7 and Ki-67 in human HCC tissues

TLR7 expression was significantly correlated with Ki-67 proliferation index (*r* = 0.3; *P* < 0.05) in the human HCC tissues (Figure 1B and 1D).

### Flow cytometry analysis of TLR7 expression in peripheral blood

**Figure 2:**
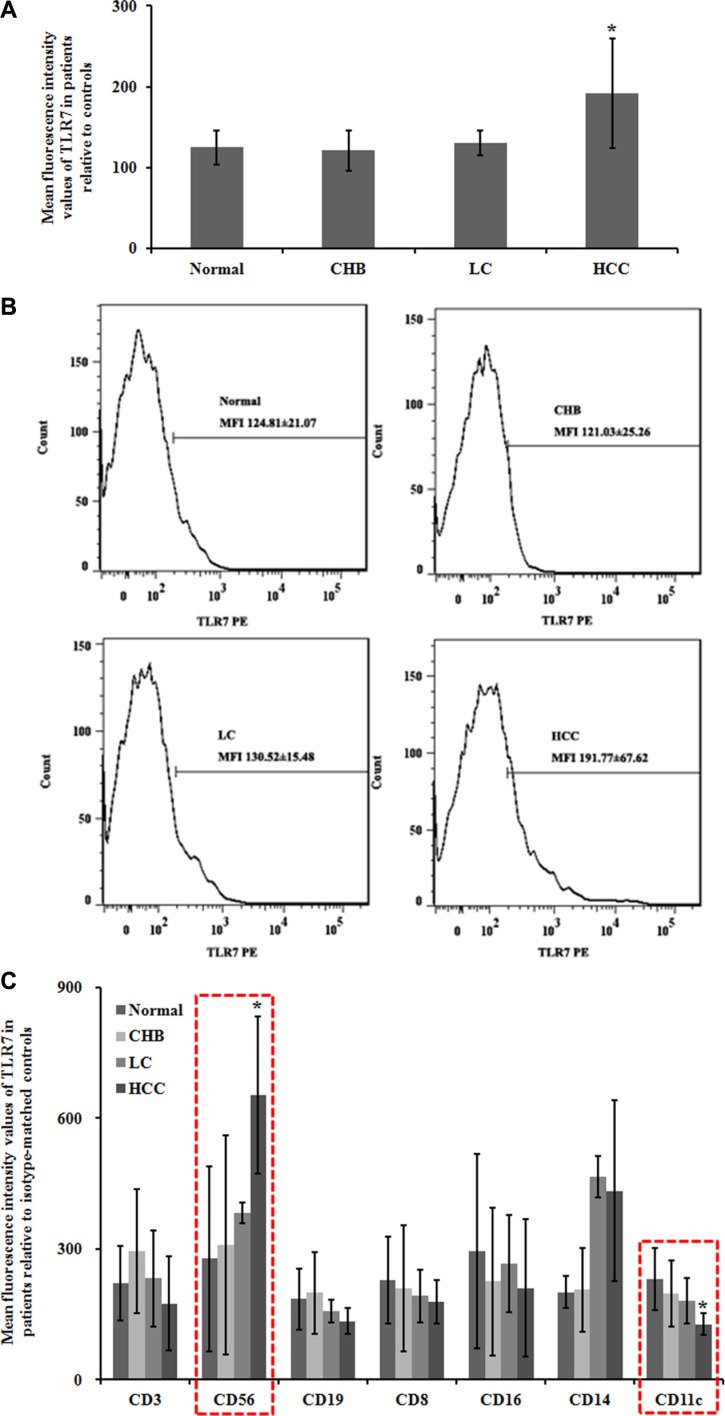
Flow cytometry analysis of TLR7 expression in peripheral blood To evaluate the efficacy of the experiment, an isotype-matched control was used. At least 3000 cells were assessed to calculate the mean fluorescence intensity values (MFI). (**A**) Statistical analyses of TLR7 MFI. Data were shown as mean ± SEM. *P < 0.05 compared with the Normal group. (**B**) Expression of TLR7 using flow cytometry analysis. (**C**) The immune cells were identified and evaluated by flow cytometry for the further observation on TLR7-related immune cell infiltrates. NK cells and pDC were in association to TLR7 expression levels. CD3, for T cells; CD8, for cytotoxic T cells; CD19, for B cells; CD56, for NK cells or NKT cells; CD16, for granulocytes; CD14, for mononuclear phagocytes; CD11c, for pDC.

To further understand pathogen-host interactions and predict development stages, we characterized TLR7 expression in peripheral blood of patients with chronic HBV infection and HBV-related LC and HCC. The mean fluorescence intensity (MFI) values of TLR7 in HCC group were significantly higher than Normal group (Figure 2A). The MFI±SEM values were (Figure 2B): Normal = 124.81 ± 21.07, CHB = 121.03 ± 25.26, LC = 130.52 ± 15.48, and HCC = 191.77 ± 67.62. Furthermore, significantly higher MFI (*P* < 0.05) of TLR7 in HCC group was observed when compared to CHB group.

Immune cell phenotypes were identified and evaluated by flow cytometry for the further observation on TLR7-related immune cell infiltrates. We found that NK cells and pDC were in association to TLR7 expression levels. The MFI ± SEM values of CD56 for NK cells were: Normal = 277.55 ± 212.21, CHB = 308.93 ± 250.96, LC = 383.33 ± 23.57, and HCC = 653.74 ± 180.43. The expression level of CD56^+^ TLR7 in HCC group was significantly higher (*P* < 0.05) than that in Normal group. The MFI ± SEM values of CD11c for pDC were: Normal = 231.31 ± 70.30, CHB = 198.25 ± 75.73, LC = 180.59 ± 52.41, and HCC = 127.33 ± 24.73. The CD11c^+^ TLR7 expression in HCC was downregulated compared with Normal group (*P* < 0.05) (Figure 2C).

### Lipid rafts are engaged in TLR7 signaling enhancement in human HCC tissues

**Figure 3:**
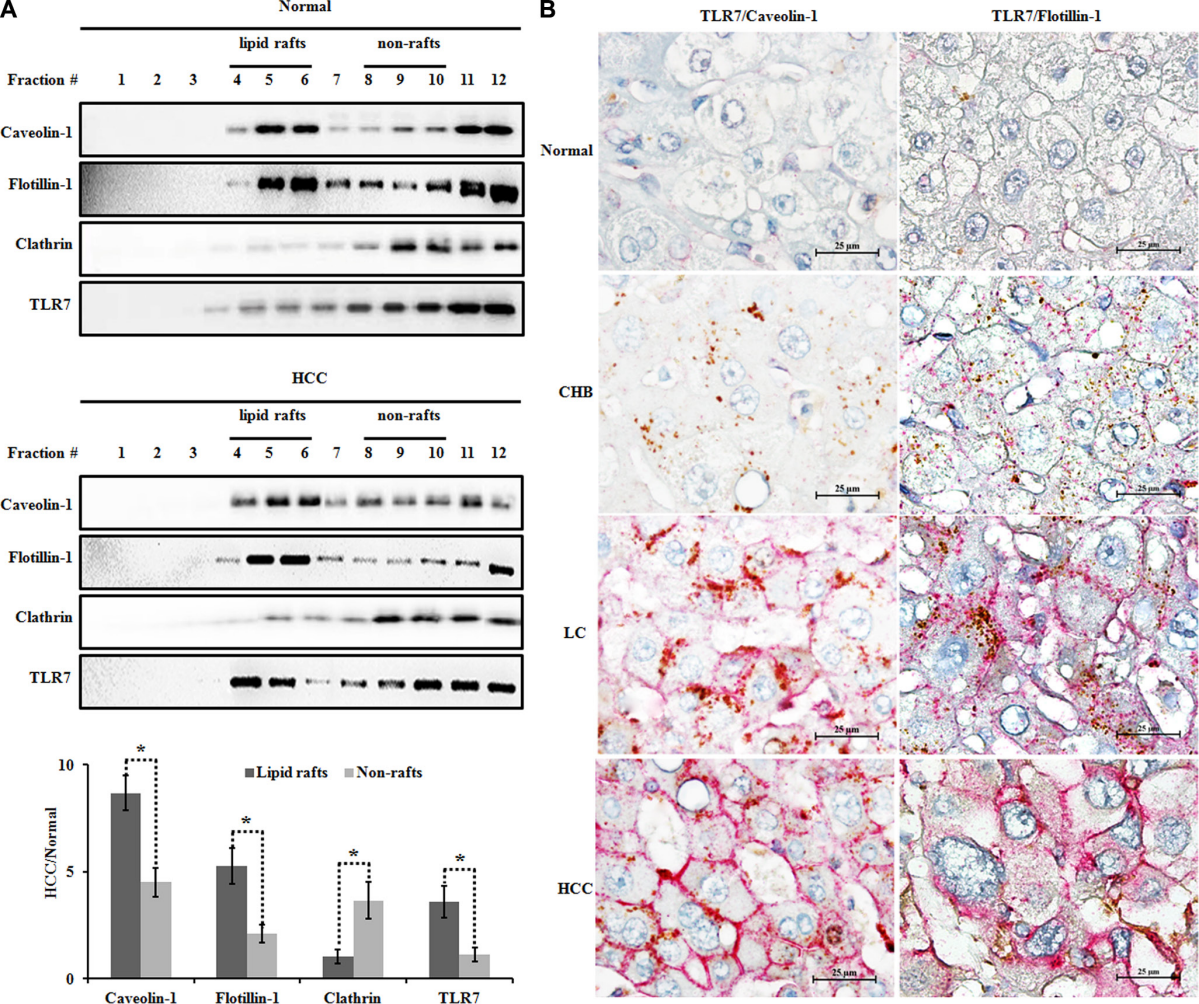
Increased TLR7 in lipid rafts of human HCC tissues (**A**) Lipid raft and non-raft microdomains were isolated using a non-detergent method and analyzed for caveolin-1 (lipid raft marker), flotillin-1 (lipid raft marker), clathrin (non-raft marker) and TLR7 distribution by western blotting. The graph showed the expression of caveolin-1, flotillin-1, clathrin and TLR7 in HCC relative to Normal tissue. *P < 0.05, lipid rafts compared with the non-rafts. (**B**) The co-expression of TLR7 and lipid rafts was analyzed by double immunohistochemistry. The co-expression of TLR7 and lipid rafts was located in the cell membrane only in HCC group but as the scattered spots in cytoplasm of live cells in Normal, CHB, LC groups. Interestingly, margination orientation of TLR7/Caveolin-1 or TLR7/Flotillin-1 from cytoplasm to membrane was observed in LC tissues. The microphotographs were magnified 1000 times.

We isolated lipid rafts and nonrafts from normal and HCC liver tissues using a simplified non-detergent isolation method [22, 24]. Lipid raft and non-raft fractions were respectively confirmed based on the relative distribution of raft markers, caveolin-1 and flotillin-1 and the non-raft marker, clathrin. Results of the distribution of lipid rafts along gradient fractions were shown in Figure 3A, in which caveolin-1 and flotillin-1 as the lipid raft resident proteins were mainly presented in fraction 5 and 6 of both tumor and Normal tissues. Furthermore, levels of caveolin-1 and flotillin-1 proteins in HCC samples elevated in fraction 5 and 6 while declined in non-raft fractions compared with Normal tissues. The expression of TLR7 was mainly located in non-raft membrane fractions in Normal tissues. Upon cancerization, a tendency toward increased translocation of TLR7 into lipid rafts was observed in HCC tissues (Figure 3A). The co-expression of TLR7 and lipid rafts was analyzed by double-labeling immunohistochemistry. Lipid raft expression of TLR7 on the plasma membrane was shown only in HCC tissues but as the scattered spots in cytoplasm of hepatocytes in Normal, CHB and LC tissues (Figure 3B). Collectively, these data suggest that hepatocarcinogenesis results in a significant increase in lipid raft contents, which concentrate and recruit more TLR7 through augmented translocation or trafficking to lipid rafts, leading to hyper-activation of downstream signaling transduction and exaggerated proinflammatory cytokine expression.

### Increased cholesterol in lipid rafts of human HCC tissues and HepG2 cells

**Figure 4:**
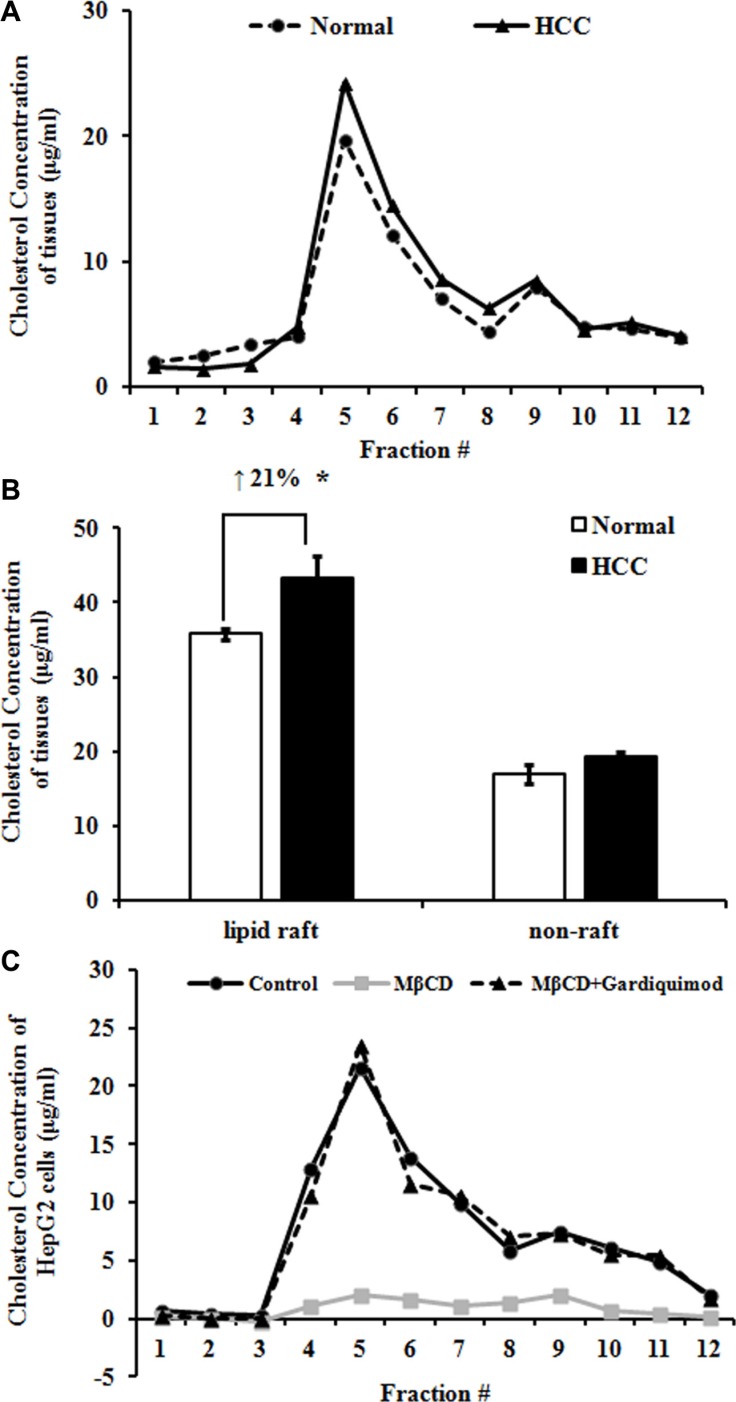
Increased cholesterol in lipid rafts of human HCC tissues and HepG2 cells (**A**) Lipid raft and non-raft fractions were isolated using a non-detergent method. Cholesterol concentration in each fraction of Normal and HCC tissues was measured with an Amplex Red cholesterol assay kit. Results were representative of at least three patients in each group. (**B**) Data from panel A were presented as raft (fractions 4–6) and non-raft (fractions 8–10) cholesterol concentration. *P < 0.05, compared with Normal. (**C**) HepG2 cells were treated with 10 mM MβCD or 1μg/ml gardiquimod for 1h and then lipid raft and non-raft fractions were pooled by centrifugation. The highest level of cholesterol was detected in fraction 5 in both HepG2 cells and tissue samples. Only MβCD depleted cholesterol, but gardiquimod resulted in an increase in lipid rafts. Results were representative of three independent experiments.

To determine why lipid raft contents increased in HCC, we isolated lipid rafts and nonrafts from Normal and HCC liver tissues using a non-detergent isolation method. We fractionated the gradient solution into 12 fractions of 1 ml per tube. Using raft markers (flotillin-1 and caveolin-1) and non-raft marker (clathrin), we designated the fractions 4–6 as lipid rafts and the fractions 8–10 as nonrafts (Figure 3A). Interestingly, we found that each lipid raft fraction contained more cholesterol in HCC group than normal group (Figure 4A), resulting in an average 21.27 ± 2.78% greater cholesterol content for the whole lipid raft fraction (i.e., fractions 4–6) compared with the same fractions from normal group (Figure 4B). On the other hand, cholesterol content was similar in non-raft fractions of normal and HCC groups (Figure 4A and 4B). Taken together, our data suggested that an increase in lipid raft content of HCC likely due to a striking increase in cholesterol content of this fraction.

To determine whether rapid alteration of cholesterol content changes lipid raft content and TLR7 expression, we incubated HepG2 cells with 10 mM MβCD or MβCD loaded with 1 μg/ml of gardiquimod for 1h and then lipid raft and non-raft fractions were pooled by centrifugation (Figure 4C). We found that the fraction 5 of HepG2 cells had the highest level of cholesterol. Only MβCD depleted cholesterol, but TLR7 agonist gardiquimod resulted in an increase of cholesterol content in lipid rafts and TLR7 expression.

### The TLR7 agonist gardiquimod promotes proliferation and migration of HepG2 cells

**Figure 5:**
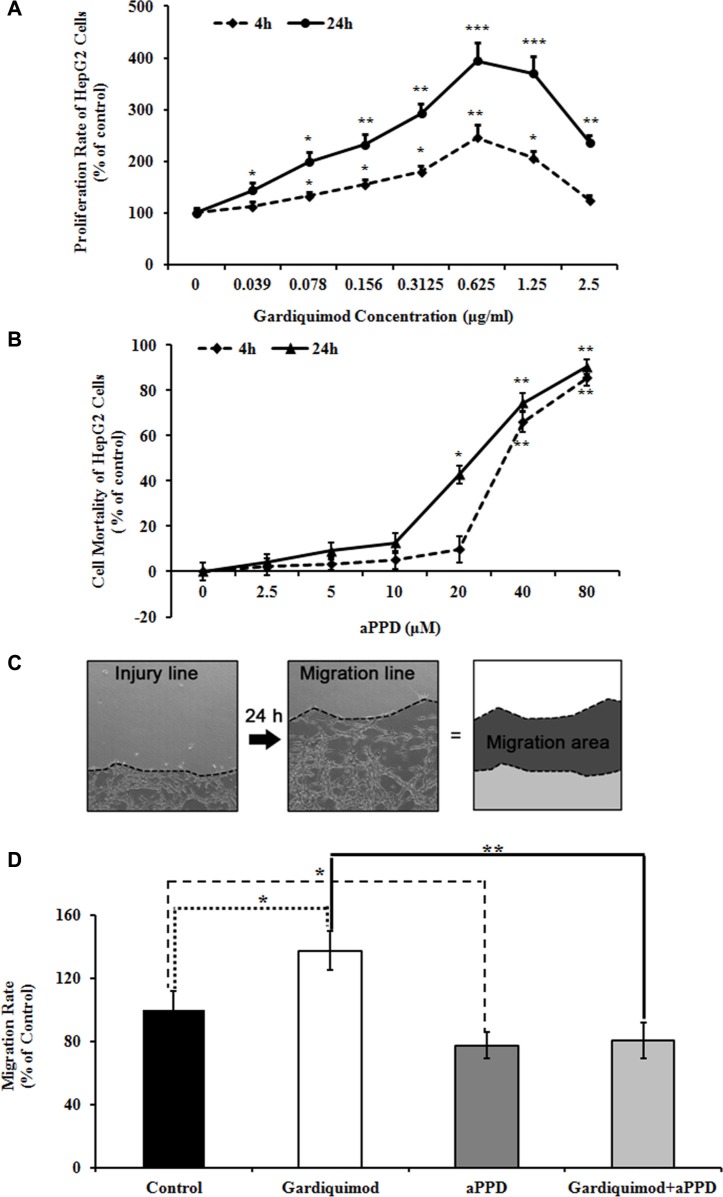
Gardiquimod promotes proliferation and migration of HepG2 cells (**A**) HepG2 cells were treated with different concentration of gardiquimod for 4 h and 24 h respectively, and the proliferation rate was detected by MTT assay. *P < 0.05, **P < 0.01, ***P < 0.001 compared to the untreated control (*n* = 3). (**B**) HepG2 cells were treated with different concentration of aPPD for 4 h and 24 h respectively, and the cell mortality was detected by MTT assay. *P < 0.05, **P < 0.01 compared to the untreated control (*n* = 3). (**C**) Migration of cells in culture dishes. The edges (dotted lines) of cultured HepG2 cells at 0 and 24 h after scratch were shown as the injury line and the migration line, respectively. The migration area was calculated as regions between the injury and migration lines. (**D**) Quantification of the cell migration rate. The migration rate of HepG2 cells was effectively reduced with the treatment of aPPD compared with gardiquimod. The data were the averages of two repeated experiments with quadruplets for each time, *P < 0.05, **P < 0.01.

To observe the effect of TLR7 agonist on hepatocyte proliferation, HepG2 cells were treated with gardiquimod for 4 h and 24 h respectively. We found that gardiquimod promoted the proliferation of hepatocytes with a greater effect at the dosage of 1.25 μg/ml, and 24 h seemed to be more efficient than 4 h (Figure 5A). We thus chose a 1μg/ml dose for subsequent experiments.

To further observe whether gardiquimod could enhance the hepatocyte migration, we measured the cell migration rates of HepG2 cells treated with 1 μg/ml gardiquimod or gardiquimod loaded with aPPD for 24 h using scratch assay *in vitro*. We also measured the cell mortality of HepG2 cells treated with different concentration of aPPD at different time points to determine the toxic dose of aPPD to the cells. There was no significant increase in the mortality of HepG2 cells with 10 μM aPPD treatment both at 4 h (5.20 ± 2.45%, *P* = 0.196) and at 24 h (12.63 ± 13.57%, *P* = 0.089). We observed a significant cell death of HepG2 cells at 24 h loaded with 20 μM aPPD (42.78 ± 14.78%, *P* = 0.002) (Figure 5B). Thus, we chose a 10 μM dose for subsequent experiments. Our previous results showed that aPPD inhibited tumor cell migration [24]. Migration rate of HepG2 cells was significantly upregulated after gardiquimod treatment in Figure 5C and 5D (137.34 ± 12.46% of control, *P* < 0.05), but effectively downregulated after aPPD treatment compared with gardiquimod treatment (80.34 ± 11.19%, *P* < 0.001).

### Enhanced lipid raft-associated TLR7 signaling results in the activation of downstream NFκB signaling pathway in HepG2 cells

**Figure 6:**
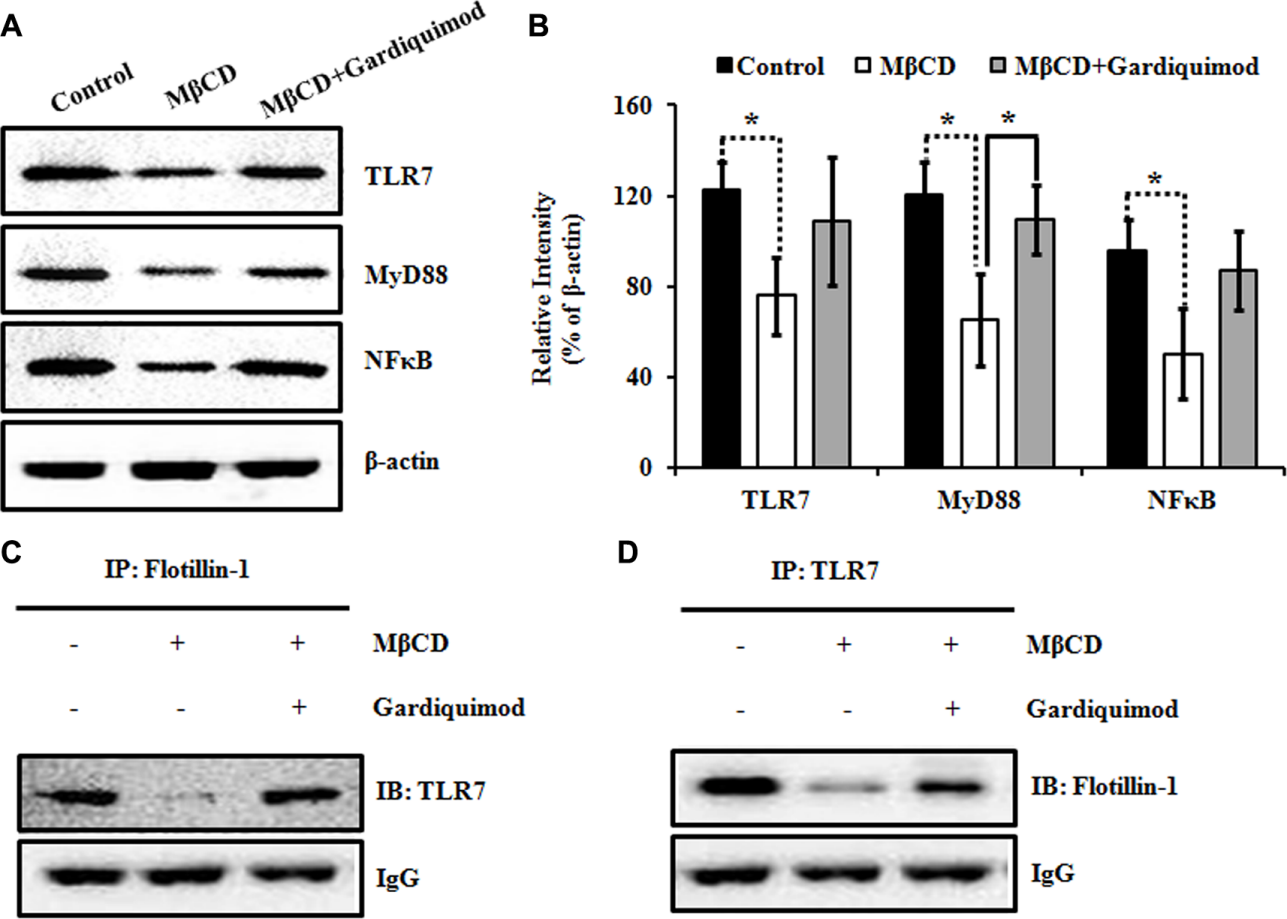
Enhanced lipid raft-associated TLR7 signaling results in the activation of downstream NFκB pathway in HepG2 cells (**A**) HepG2 cells were incubated with 10 mM MβCD or 1 μg/ml gardiquimod for 1 h. Cell lysates were harvested and the proteins were analyzed by immunoblotting with the indicated antibodies. (**B**) TLR7, MyD88, NFκB were quantified and normalized to β-actin. Data were presented as mean ± SEM; *n* = 3. Statistically significant differences between groups were indicated (*P < 0.05). (**C**) HepG2 cells were treated with 10 mM MβCD or 1 μg/ml gardiquimod for 1h and then lipid rafts (fractions 4–6) were pooled, and immunoprecipitated with anti-flotillin-1 polyclonal antibody, followed by immunoblotting with anti-TLR7 monoclonal antibody. Results were representative of three independent experiments. (**D**) Lipid rafts isolated as described in panel C were immunoprecipitated with anti-TLR7 monoclonal antibody, followed by immunoblotting with anti-flotillin-1 polyclonal antibody. Results were representative of three independent experiments.

TLR7 was shown to translocate from the endoplasmic reticulum to the endosome upon stimulation by specific agonists, resulting in activation of NFκB pathway [13]. To determine whether the downstream NFκB pathway is upregulated in gardiquimod-treated HepG2 cells, we stimulated HepG2 cells with 10 mM MβCD or MβCD loaded with 1 μg/ml gardiquimod for 1h before isolating cell lysates for immunoblotting. As shown in Figure 6A and 6B, we observed TLR7, MyD88 and NFκB content were significantly declined in MβCD-treated cells (*P* < 0.05), but a trend of increased content in gardiquimod-treated cells, especially the MyD88 content (*P* < 0.05). Because gardiquimod stimulation was shown not only to induce trafficking of TLR7 to the endosome but also to induce translocation of TLR7 to lipid rafts, we then tested the hypothesis that gardiquimod may induce more TLR7 translocation to lipid rafts. Next, we pooled lipid raft (fractions 4–6) fractions isolated from MβCD ± gardiquimod treatment and performed immunoprecipitation (Figure 6C and 6D) to visualize and quantify the translocation of TLR7 to lipid rafts. We found that gardiquimod stimulation significantly triggered increased translocation of TLR7 to lipid raft fractions in gardiquimod-treated cells vs. MβCD-treated cells. These combined results suggest that the appreciably increased lipid raft content increases MyD88-dependent activation of plasma membrane and intracellular TLR7 through augmented translocation or trafficking to lipid rafts, resulting in activation of downstream NFκB pathway and exaggerated pro-inflammatory cytokine expression.

## DISCUSSION

Conclusions can be drawn from the above results that (1) the expression of TLR7 is obviously increased in HCC patients and in HepG2 cells. Importantly, patients with viral hepatitis or cirrhosis rarely express TLR7; (2) TLR7 activation is involved with marked increase in tumor cell proliferation and migration; (3) lipid rafts are positively associated with enhanced TLR7 signaling in HCC; (4) an increase in lipid raft content of HCC is likely attributable to a striking increase in cholesterol accumulation; (5) downstream NFκB pathway is involved with enhanced raft-associated TLR7 signaling in HCC. These results indicate that TLR7 may be a target for the treatment of HCC in future. Inhibiting TLR7 with antagonists, like aPPD, becomes a focus for their potential use as a novel therapeutic approach for HCC based on the results of our study.

TLRs are usually involved in innate immune response and TLR signaling is closely related to various chronic liver diseases. Especially, TLR7 is excessive expression in many malignancies [30–32]. However, there is little research on the role of TLR7 in HCC. In the present study, TLR7 expression was higher in human HCC cases than that with viral hepatitis, cirrhosis and Normal controls. Mohamed *et al.* had previously reported the high expression of TLR7 in HCC patients [14]. In their study, TLR7 tended to gather in the perinuclei of hepatoma cells. However, we found that TLR7 was mainly expressed in the cell membrane and perinuclei. The unusual localization within HCC cells may be related to the mitogenic activity of TLR7 contributing towards hepatocarcinogenesis. Then the effect of activating TLR7 was further confirmed in human HCC cell line using a TLR7 agonist gardiquimod, which can substantially induce cell proliferation and migration. On the one hand, overactive TLR signal provides a microenvironment that is necessary for malignant cell proliferation and migration. On the other hand, TLR antagonists can also be used to inhibit cancer cell growth. This leads to the further understanding that the activation of TLRs acts as a “double-edged sword” in the progression or treatment of cancer. Unlike results from our studies, some papers showed that TLR7 expression down-regulated in HCC [7, 33]. Lin et al. found the down-regulated expression of TLR7 through cancerous and non-cancerous liver tissues from HBV/HCV-related HCC [7]. We studied the expression of TLR7 in normal liver tissues by resecting parts of liver from hepatic cyst, calculus of intrahepatic duct, gallbladder polyps et al. and HBV-related HCC. Most of adjacent non-cancerous tissues had cirrhosis background, so the expression of TLR7 may differ in normal livers and non-cancerous liver tissues.

The main finding in this study was on TLR7 mediated mechanism of HCC development and progression via lipid rafts on the cell membrane. Lipid rafts are liquid-ordered membrane microdomains enriched in highly ordered saturated sphingolipids and cholesterol, which are conducive to lateral movement. Lipid rafts are not only considered as a means to explain the spatial segregation of certain signaling pathways deriving from the cell surface, but also provide the necessary microenvironment so that certain specialized signaling events can take place, such as the innate immune recognition. TLRs as the crucial “sensing” apparatus of the innate immune system are recruited into lipid raft microdomains in response to ligands. Subsequently, sphingolipids, cholesterol and TLRs coalesce together and form the nanoscale assemblies as signaling platforms to stabilize and transduce signals that lead to innate immune activation. Our previous study suggested that the two typical lipid raft resident proteins, namely, caveolin-1 and flotillin-1were overexpressed in HCC compared to adjacent noncancerous tissues [22]. The present study has found direct evidence for the involvement of lipid rafts in the relationship of enhanced TLR7 signaling and the occurrence and progression of HCC by increasing cholesterol enrichment in lipid rafts. With these findings in mind, we hypothesized that the increased lipid raft content in HCC might result in enhanced TLR7 trafficking into rafts and our results appropriately supported this hypothesis (Figures 4 and 6).

Because the membrane-bound TLRs share many properties and similar activation mechanisms, we assume that the occurrence and progression of HCC lead to increased translocation or recruitment of all MyD88-dependent TLRs into lipid rafts, which results in the constitutive activation of NFκB to produce augmented proinflammatory cytokines and promote cell proliferation and survival. Previous reports have shown that lipid rafts are vital to the inflammatory cytokine-mediated NFκB activation. For instance, the activation of TNF-α results TNFR in translocating into lipid rafts, and disrupting lipid rafts or interfering with the lipid raft composition could prevent TNF-α-associated TNFR from trafficking into lipid rafts and reduce the enrollment of RIP into the engaged receptor, indicating that lipid raft organization is essential to TNF-α-induced NFκB stimulation [16, 34]. In accordance with these previous reports, we found that an increase in cholesterol accumulation led to the hyper-expression of caveolin-1 and flotillin-1, consequently increased the number of lipid raft microdomains, whereas depletion of cholesterol disrupted lipid raft formation. Therefore, cholesterol may promote the formation of lipid rafts and facilitate the recruitment of TLR7 and its ligands to lipid rafts, thus contributing to constitutive NFκB activation. This is one of the many pieces of supportive evidence for the hypothesis that TLR-driven inflammation is linked with furthering malignant development. In an ever-growing field, more research in the TLR signaling pathways have reflected about our understanding of the involvement of TLRs in cancer [13, 14, 27].

Our previous study demonstrated that aPPD, the final metabolite of ginseng saponin, was a high efficient breaker for lipid rafts. Unlike MβCD, aPPD altered the contents of resident proteins in the lipid rafts rather than cholesterol content [24]. Because the structure of aPPD is more similar to cholesterol, it would not be surprising if it functions as a stronger raft disruptor through intercalating itself into the lipid rafts to bring about changes in the microenvironment of the membrane, which in turn causes an alteration of the protein constituent of lipid rafts. Our results demonstrated that aPPD inhibited HepG2 cell proliferation and migration. More interestingly, the migration rate of HepG2 cells induced by gardiquimod (a TLR7 agonist) was efficiently decreased after aPPD treatment (Figure 5). These findings suggested that aPPD may serve as a potent TLR7 antagonist for pharmacological intervention in tumor therapy.

In conclusion, certain viral products, as TLR ligands, anchoring in the cell membrane directly activate TLR7 signaling. Once the binding of TLR7 and ssRNA, the polymer subsequently aggregates in lipid raft microdomains at the endosomal membrane and induces the intracellular signaling cascades which contribute to activation of NFκB and production and secretion of proinflammatory cytokines that promote the proliferation and formation of cancerous cells. To our knowledge, this study is the first to provide new insights into the pathogenesis of HCC that lipid rafts promote liver cancer cell proliferation and migration by up-regulation of TLR7 expression on HCC patients and cell lines. In summary, our data suggest that inhibition of the signaling pathway involved raft-associated TLR7 by using antagonists could serve as a novel approach to prevent the occurrence and progression of HCC.

## MATERIALS AND METHODS

### Materials

Antibodies to TLR7, caveolin-1, flotillin-1, MyD88, clathrin, and Ki-67 were purchased from Abcam, Inc. (Cambridge, MA, USA). NFκB antibody was purchased from Cell Signaling Technology, Inc. (Boston, MA, USA). β-actin antibody, HRP-conjugated anti-rabbit, and anti-mouse IgG were purchased from CWBio, Inc. (Beijing, CN). Mouse IgG_2A_ anti-Human TLR7 PE-conjugated monoclonal antibody and mouse IgG_2A_ PE-conjugated isotype control were purchased from R&D Systems, Inc. (Minneapolis, MN, USA). BV510 anti-human CD14 antibody and BV421 anti-human CD16 antibody were purchased from BD Biosciences (San Jose, CA, USA). APC/Cy7 anti-human CD3 antibody, PerCP/Cy5.5 anti-human CD8a antibody, FITC anti-human CD56 antibody, PE/Cy7 anti-human CD11c antibody and APC anti-human CD19 antibody were purchased from Biolegend (San Diego, CA, USA). Protease inhibitor cocktails were obtained from Roche Molecular Biochemicals (Mannheim, DE). BCA protein assay kit and PVDF membranes were purchased from Bio-Rad (Hercules, CA). MβCD, media (Dulbecco's modified Eagle's medium, DMEM), MTT and other chemicals were purchased from Sigma (St. Louis, MO, USA). Gardiquimod was purchased from Santa Cruz (California, USA). aPPD was provided by Shanghai Innovative Research Centre of Traditional Chinese Medicine (Shanghai, China). The compound was 97.9% pure as measured by HPLC analysis.

### Patients

Human liver tissues (formalin-fixed paraffin-embedded tissues and cryostat tissues) and peripheral blood were used in this study. The study included patients with chronic hepatitis B (CHB) (*n* = 23), HBV-related liver cirrhosis (LC) (*n* = 18), HBV-related HCC (*n* = 68) and normal liver (*n* = 21). The patients were enrolled at Beijing 302 Hospital (Beijing, China) from January 2014 to December 2014. The study was conducted according to the guidelines of the Declaration of Helsinki. Informed consent was obtained from each patient, and the study protocol was approved by the Ethics Committee of Beijing 302 Hospital. The clinical and pathological characteristics of the patients are listed in Table 1.

The diagnostic criteria conformed to “The guideline of prevention and treatment for chronic hepatitis B” [35]. All LC patients were in Child class B and C by Child-Pugh score evaluation. HCC patients were confirmed by liver tissue biopsy. All normal were HBsAg-negative patients with other liver diseases.

### Cell cultures and treatments

Human HepG2 cells were cultured in DMEM supplemented with 10% FBS, 100 U/ml penicillin and 100 mg/ml streptomycin, at 37°C in a humidified atmosphere containing 5% CO_2_ and fed every 2–3 days. A stock solution of 50 mM aPPD was prepared in 100% ethanol and diluted to proper concentrations in DMEM immediately before each experiment. Drug-treatment cells were pretreated with DMEM for 4 h at 37°C, followed by the addition of 10 mM MβCD, 1 μg/ml gardiquimod or 10 μM aPPD for 1 h.

### Lipid raft isolation

The fresh liver tissue samples or HepG2 cells in five 100-mm dishes were mixed with lysis buffer (150 mM NaCl, 20 mM Na_2_HPO_4_, 2 mM NaH_2_PO_4_, 20% v/v glycerol, 2 mM sodium orthovanadate with protease inhibitors, pH 7.4) and homogenized 30 times with a tight Dounce homogenizer (Sigma). Samples were further disrupted by intermittent sonication (six 30s pulses with a 1min cooling period in between) and then centrifuged at 10 K rpm (Beckman-Coulter Optima L-90 K ultracentrifuge with a SW55Ti rotor, CA, USA) for 10 min at 4°C to separate cell debris and nuclear materials. The supernatant was then centrifuged at 32.5 K rpm (SW55Ti rotor) for 90 min at 4°C to pellet the plasma membrane (PM). The PM was suspended and solubilised in 2 ml solubilising buffer containing 0.5% v/v Triton X-100 in Mes-buffered saline (MBS: 25 mM Mes, 0.15 M NaCl, pH 6.5), protease inhibitors and 2 mM sodium orthovanadate for 15min on ice. Then, 2 ml of solubilised PM were further diluted with an equal volume of 80% sucrose in MBS and loaded on the bottom of a 13 ml ultracentrifuge tube overlaid with 4 ml of 30% sucrose/MBS. Finally, 4 ml of a 5% sucrose/MBS solution were added as the top layer of the gradient. The gradient was centrifuged at 31 K rpm (SW41Ti rotor) for 16 h at 4°C to isolate the lipid raft and non-raft compartments. The gradient was then fractionated into 12 fractions [22, 24].

### Immunoblotting and immunoprecipitation

Western blotting was performed using specific antibodies against: TLR7, caveolin-1 (as lipid raft marker), flotillin-1 (as lipid raft marker), clathrin (as non-raft marker), MyD88, NFκB, β-actin. Flotillin-1 was immunoprecipitated with a flotillin-1 polyclonal antibody from treated HepG2 cells, followed by immunoblotting with antibody against TLR7. A Pierce co-immunoprecipitation kit (Thermo Scientific, Rockford, lL, USA) was used according to the manufacturer's protocol. TLR7 was immunoprecipitated with a TLR7 monoclonal antibody and then immunoblotted with antibody against flotillin-1. Blots were developed using HRP-linked secondary antibody. Signals were detected using enhanced chemiluminescence (Perkin-Elmer Life Sciences) and band intensities were quantified using Image J software (NIH, Bethesda, MD, USA).

### Immunohistochemistry and double-labeled immunohistochemistry

For selected cases, formalin-fixed, paraffin-embedded liver tissues were characterized at the time of diagnosis by immunohistochemistry using the following antibodies: TLR7 rabbit polyclonal (1:150 dilution), Ki-67 rabbit monoclonal (1:100), caveolin-1 mouse monoclonal (1:300) and flotillin-1 mouse monoclonal (1:300). Stains were performed on an automated stainer using heat induced epitope retrieval (CC1 standard, Ventana Benchmark) and 3, 3′- diaminobenzidine detection (DAB, Ventana). Double-labeling immunohistochemistry was performed using an antibody cocktail for TLR7 rabbit polyclonal (1:150 dilution) and caveolin-1 mouse monoclonal (1:300) or flotillin-1 mouse monoclonal (1:300) using DAB detection for TLR7 and fast red for detection of caveolin-1 or flotillin-1 (Ventana).

The TLR7 staining was evaluated semi-quantitatively by two independent pathologists using the scoring system described in Table 2. Proliferation index was calculated for each HCC by counting the number of tumor cells positively staining for nuclear Ki-67 from a total of 1000 tumor cells (expressed as a percentage).

**Table 2:** TLR7 immunohistochemistry scoring system

Staining score	Description
0	No staining or (+) membranous staining intensity in less than 1/3rd of cells
1	(+) membranous staining intensity in more than 1/3rd of cells or (++) intensity in less than 2/3rd of cells
2	(++) or (+++) membranous staining intensity in more than 2/3rd of cells

### Flow cytometry

A total of 6 × 10^6^ − 10 × 10^6^ peripheral blood was collected. The cells were then stained with mouse IgG_2A_ anti-human TLR7 PE-conjugated monoclonal antibody, mouse IgG_2A_ PE-conjugated isotype control, BV510 anti-human CD14 antibody, BV421 anti-human CD16 antibody, APC/Cy7 anti-human CD3 antibody, PerCP/Cy5.5 anti-human CD8a antibody, FITC anti-human CD56 antibody, PE/Cy7 anti-human CD11c antibody and APC anti-human CD19 antibody. Stained cells were analyzed on a Canto^™^ II machine (BD Biosciences, San Jose, CA, USA). Data was analyzed using FlowJo 7.6.1 software (Stanford University, Stanford, CA, USA).

### Cholesterol measurement

Fifty microliters of each fraction were analyzed with an Amplex Red cholesterol assay kit (Molecular Probes, Eugene, OR, USA) according to the manufacturer's protocol.

### Cell proliferation assay

Proliferation was determined by culturing 2.5 × 10^4^/well of human HCC line HepG2 with different doses of gardiquimod and aPPD in 96-well plates for 4 h and 24 h, one day before experiments. 3-[4, 5-dimethylthiazol-2-yl] -2, 5-diphenyltetrazolium bromide (MTT) (50 μl, 0.5 mg/ml) was added 4 h before the end of the incubation period. Dimethylsulfoxide was used to dissolve granules, and the absorbance at 570 nm of each sample was determined with a microplate autoreader (BioTek, Synergy H4, USA). The proliferation rate was determined as follows: (A of treatment group/A of PBS group) × 100%.

### Cell migration assay

HepG2 cells were cultured in six-well culture dishes to 70–90% confluence, followed by scratch with 1 ml sterilized pipette tips. After washing with pre-warmed PBS, cells were cultured in serum-free DMEM for 24 h. Images were taken at 0 h and 24 h after scratch by Olympus FV1000 confocal microscope (Olympus, Beijing, China). Images of same spots taken at 0 h and 24 h were overlaid and adjusted with Photoshop CS2 software (Adobe, China). The edge of cultured cells was marked by linking cell bodies of furthest, but continually, related cells from unscratched regions. The edge at 0 h and 24 h were defined as the injury line and migration line, respectively. The area between injury and migration lines was considered as the migration area and was subject to quantification [24]. Areas of migration on images were measured with Image J.

### Statistical analysis

Continuous variables were analyzed with Students' *t*-test or ANOVA in case of normal distribution. For intergroup comparison, Mann-Whitney *U*-test was used for the non-parametric analyses and chi-square test. For correlation, Spearman rank correlation was used.
